# Impaired lung function is associated with elevated blood biomarkers of AD/ADRD: Unraveling the interplay with risk of dementia

**DOI:** 10.21203/rs.3.rs-8311583/v1

**Published:** 2025-12-30

**Authors:** Sithara Vivek, Eileen M Crimmins, Jung Ki Kim, Jessica Faul, David R Jacobs, Weihua Guan, Bharat Thyagarajan

**Affiliations:** University of Minnesota; University of Southern California; University of Southern California; University of Michigan; University of Minnesota; University of Minnesota; University of Minnesota

**Keywords:** Lung function, Dementia, AD biomarkers, Older adults, Mechanism

## Abstract

**Background and Objectives:**

Impaired lung function (ILF) has been associated with cognitive decline and dementia risk in multiple cohorts, yet the role of circulating Alzheimer disease (AD) biomarkers in this relationship is not well understood. We aim to assess the associations between ILF and AD biomarkers and to determine whether these biomarkers mediate the relationship between ILF and incident dementia.

**Methods:**

Serum p-Tau181 and plasma Aβ42/40, NfL, and GFAP were measured in 4,072 participants (mean age 66 ± 10; 59% women) in the 2016 Health and Retirement Study. Peak Expiratory Flow (PEF) was assessed in 2012/2014, and cognitive function was measured at four time points between 2014 and 2020 (every two years) to determine dementia status. Impaired lung function (ILF) was defined as predicted PEF <80%. Multivariable regression examined associations between lung function and AD biomarkers; causal mediation analysis evaluated their role in linking lung function to incident dementia.

**Results:**

In total, 881 (21.6%) participants had ILF and 272 (6.8%) participants developed dementia. After adjusting for demographics, education, BMI, smoking, comorbidities, inflammation, eGFR and *APOE e4*, ILF was associated with a higher risk of dementia (HR=1.74; 95% CI (1.34, 225)). Individuals with ILF had 0.10 SD higher NfL (SE= 0.03; p= 0.004) and 0.09 SD higher p-Tau 181 (SE= 0.03; p= 0.002) compared to those without ILF. NfL mediated 7.3% (p=0.01) of the total effect of ILF on dementia, while p-Tau 181 mediated 5% (p=0.05) of this association.

**Discussion:**

ILF was associated with elevated levels of neurodegeneration markers NfL and p-Tau 181, which partially mediated its relationship with dementia risk. These findings highlight the importance of monitoring blood protein biomarkers in individuals with impaired lung health to facilitate early interventions.

## BACKGROUND

Extensive research across diverse populations has consistently shown that reduced lung function is associated with cognitive decline and an increased risk of dementia^1–7^. Maintaining optimal lung health may therefore play a critical role in preserving cognitive abilities. Studies with extended follow-up data reported that higher lung function measures - such as FEV_1_, FVC and FEV_1_/FVC ratio - were associated with a slower rate of cognitive decline across multiple domains, including memory, language, and processing speed/attention^7,8^. A study in the Atherosclerosis Risk in Communities (ARIC) cohort showed that lung disease, including both restrictive and obstructive lung diseases, among middle-aged adults was associated with increased risk of developing dementia later in life^9^. Additionally, a recent study in the National Health and Aging Trends Study (NHATS) cohort, a nationally representative sample of older adults in the US, found that higher levels of lung function measured by peak expiratory flow (PEF) was associated with lower risk of developing dementia, exhibiting a dose-dependent relationship^6^. Emerging evidence suggests that impaired lung function (ILF) may contribute to poor cognitive outcomes through both neurodegenerative and vascular pathways. In the Rush Memory and Aging Project (MAP), poor pulmonary function was associated with pathological features of Alzheimer’s disease (AD) -such as global AD pathology, amyloid beta (Aβ) load, and neurofibrillary tangles- as well as markers of cerebral vascular disease^10^. Recent meta-analyses^11^ further support this hypothesis by demonstrating associations between ILF and brain imaging biomarkers of neurodegeneration, vascular injury and AD pathology. However, it remains unclear whether circulating AD biomarkers–now widely used as early indicators of neurodegeneration and pathological change–contribute to this association.

Ultrasensitive protein assays now enable detection of AD-related proteins from small blood samples, and these minimally invasive biomarkers shown promise in early identification of cognitive decline and in staging of AD/ADRD^12–14^. Several key protein biomarkers measured in plasma and serum, including Amyloid beta 42/40 ratio^15^, Neurofilament Light Chain (NfL)^16^, Glial Fibrillary Acidic Protein (GFAP), and phosphorylated tau (p-Tau 181 and 217^12,17^) were associated with decline in cognitive function and risk of AD/ADRD^16^. Plasma p-Tau proteins have emerged as a promising candidate marker during symptomatic and preclinical AD when it is used with Aβ42/Aβ40^13^. A recent case-control study demonstrated the promise of using all plasma biomarkers and *APOE e4* for prediction of AD clinical diagnosis that reached area under receiver operating characteristic curve (AUC) = 0.81^18^. Increasing research in blood-based biomarkers has demonstrated the clinical utility of these AD protein biomarkers for risk stratification and targeted interventions. Improving the validity of AD/ADRD biomarkers is crucial for improving early diagnosis and potentially developing treatments for this condition^13,19^. This requires identifying factors that influence biomarker concentrations. For example, a recent study demonstrated the need for accounting for renal function and obesity in the analysis of NfL and GFAP^20^. In Multiple Sclerosis patients, higher BMI was inversely associated with circulating NfL^21^ and GFAP^22^ levels. However, the role of lung function in influencing these biomarkers remains unclear.

We hypothesized that impaired lung function is associated with higher levels of circulating AD/ADRD protein biomarkers and that these biomarkers mediate the association between impaired lung function to the higher risk of dementia in older adults. To test this, we (1) evaluated the association between PEF and circulating levels of AD biomarkers (Aβ 42/40 ratio, p-Tau 181, NfL, and GFAP) and, (2) investigated the role of AD biomarkers mediating the association between impaired lung function (ILF) and risk of dementia in a nationally representative sample of older adults in the Health and Retirement Study (HRS).

## METHODS

### Study population

**Figure 1:**
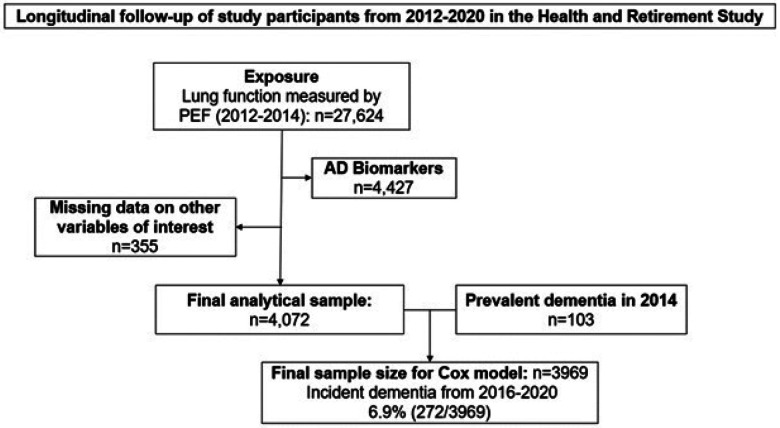
Study design and timeline: In the Health and Retirement Study (HRS) cohort, we identified participants who have lung function measure available in 2012/2014 biennial surveys and blood AD biomarkers measured in 2016 survey and cognitive function measures available from 2014 – 2020 every two years.

Health and Retirement Study (HRS) is a biennial survey of older adults in the United States that started in 1992 based on a multi-stage area probability design involving geographical stratification and clustering and oversampling of certain demographic groups and collects a wide-range of data on health, biomarkers, genetics, employment, wealth and family^23^. HRS follows participants longitudinally until death and employs a steady-state design to replenish the sample with new participants to maintain population representativeness as the study sample has aged. Additional details of the HRS study design and measurements can be found in previous publications^24–26^. We analyzed data from a subsample of individuals (n=4427) who participated in the 2016 HRS Venous Blood Study (VBS)^27^ and had AD biomarker assessments. The final analytic sample for the primary analysis comprised of 4072 individuals after excluding those missing data on exposure, outcomes or covariates are shown in Figure 1.

The HRS has been approved by the Health Sciences and Behavioral Sciences Institutional Review Board at the University of Michigan. Informed consent was obtained from all respondents in the HRS.

### Exposure measurement: Peak Expiratory Flow (PEF)

In the HRS, trained interviewers employed a standardized assessment of lung function using a peak flow meter, measuring how much air a person can exhale in one breath and reporting the measure as peak expiratory flow (PEF) in L/minute^28^. The assessment was repeated three times. We used the highest of three PEF readings from the 2012 or 2014 HRS in-person visits, as physical measures were collected from a random half-sample in 2012 and the remaining half in 2014 due to the biennial data collection design. Subsequently, we estimated the percent predicted PEF using Hankinson’s equation^29^, which accounts for individual characteristics including age, sex, race and height. We classified participants as having ‘Impaired lung function (ILF)’ if their percent predicted PEF was less than 80% based on the baseline 2012/2014 measure. To assess lung function decline, we calculated the 4-year change in percent predicted PEF from the 2012/2014 to 2016/2018 measures.

### Blood-based AD protein biomarkers

AD protein biomarkers were measured in a probability sample drawn from HRS participants in the 2016 Venous Blood Study (VBS). This included individuals aged 60 and older eligible for the 2016 Harmonized Cognitive Assessment Protocol (HCAP), as well as a random half-sample of participants under age 65 who are expected to be eligible for a future HCAP^27^. The Simoa Human Neurology 4-Plex E (N4PE) assay (Quanterix Inc., Billerica, MA) was used to measure levels of three biomarkers from plasma samples, amyloid beta 42/ 40 ratio(Aβ42/40), Glial Fibrillary Acidic Protein (GFAP), and Neurofilament light (NfL). Serum was used to assay p-Tau 181. Sample preparation and assays were performed at the University of Minnesota in the Advanced Research Diagnostics Laboratory (ARDL) based on the protocol previously validated^30^.

### Incident dementia

Cognitive function was assessed in the HRS every 2 years from 2014 to 2020. A composite score of overall cognitive performance consisted of scores from four tests: immediate and delayed 10-noun word recall, serial 7-subtraction test, and a backward count from 20. Based on previously published work, we employed the Langa-Weir classification algorithm^24^ to define dementia based on the 27-point cognitive function scale. Participants scoring between 0 and 6 on the 27-point scale were classified as having Dementia, those scoring between 7 and 11 as having Cognitive impairment no dementia (CIND), and those scoring between 12 and 27 as Normal. After excluding participants with dementia in the 2014 survey, we estimated incident dementia among those with Normal or CIND status, using cognitive test scores from the 2016, 2018, and 2020 surveys. Follow-up time ranged from 6 to 8 years, depending on whether lung function was measured in 2014 or 2012, respectively.

### Covariates

Demographic characteristics at baseline–including age, sex, race/ethnicity (White, Black, Hispanic, and Other), smoking status (current, former, or never smoker), and years of education–were collected during the 2012/2014 core survey. Body mass index (BMI) was calculated using measured height and weight from 2012/ 2014 surveys or self-reported values if measured height and weight were not available. BMI (kg/m^2^) was calculated using the equation weight (pounds)/ (height * height (inches)) * 703. We estimated comorbidity index by counting the number of self-reported chronic conditions such as type 2 diabetes, cancer, hypertension, stroke, heart condition, arthritis and psychiatric problems. We calculated estimated glomerular filtration rate (eGFR) using the new CKD Epi race-free equation based on serum levels of creatinine and cystatin C measures in the 2016 VBS^27^. An inflammatory latent variable was estimated using a confirmatory factor analysis to represent systemic inflammation based on C-reactive protein (*hsCRP*), neutrophil to lymphocyte ratio (NLR) and Cytokines (*IL-6*, *IL-10*, *IL-1RA*, *IGF1*, and *sTNFR-1*) measured in the 2016 VBS^31^. Additionally, we adjusted for *APOE* ε4 allele status, a genetic risk factor for Alzheimer’s disease (AD), determined by the TaqMan assay, with carriers defined as individuals possessing one or two ε4 alleles^32,33^.

### Statistical analysis

AD biomarkers were log-transformed to address skewed distributions and then standardized to facilitate comparability with other cohorts in statistical analyses. We standardized the % predicted PEF in 2012/2014 and the change in PEF from 2012/2014 to 2016/2018, so that one unit corresponds to one standard deviation. We used ANOVA tests for continuous variables and chi-square tests for categorical variables to determine differences in participant characteristics across ILF and normal PEF groups. Multi-variable linear regression models were used to determine the association of baseline ILF (2012/2014) and decline in lung function (from 2012/2014 to 2016/2018) with each continuous measure of AD biomarkers in the 2016 survey. Models were adjusted for age, sex, race and ethnicity, years of education, body mass index, smoking status, comorbidity index, kidney function (eGFR), systemic inflammation and *APOE* ε*4* allele status.

We used Cox proportional hazards regression model to estimate association between ILF and risk of dementia and reported hazard ratios and 95% CI over six years of follow-up. For participants who did not develop dementia, follow-up time was censored at their last assessment in the 2020 core survey. Causal mediation analysis was performed to assess the mediating role of AD biomarkers in linking the association between ILF and risk of dementia using the *causalmed* procedure in SAS. All statistical analyses were performed in SAS v9.4 (SAS Institute, Inc., Cary, NC).

## RESULTS

Among 4072 participants in the study, 58.9% were women (n=2397), with mean (±SD) age of 66.2 (±10.3), 21.6% (n=881) had ILF (% predicted PEF < 80%) and 2.5% (n=103) had prevalent dementia in 2014 and 6.9% (n=272) developed dementia over 6 years of follow-up. Black, Hispanic individuals and current smokers had a higher prevalence of ILF at baseline (Table 1).

### Association between lung function and blood biomarkers of AD

We found that lower baseline percent predicted PEF, modeled as a continuous predictor, was significantly associated with higher concentrations of p-Tau181, NfL, and GFAP after adjustment for all covariates (Table 2A). Each 1-SD higher percent predicted PEF was associated with lower levels of p-Tau181 (β = −0.04, p = 0.004), NfL (β = −0.05, p < 0.001), and GFAP (β = −0.04, p = 0.001). No significant association was observed with the Aβ42/40 ratio. In fully adjusted models, participants in the lowest PEF quartile had the highest biomarker levels, showing a graded inverse relationship across quartiles (Table 2A). When using impaired lung function (ILF; PEF <80%) as a binary predictor, multivariable-adjusted linear regression models showed that individuals with ILF had significantly higher levels of p-Tau181 (β = 0.10, p = 0.0040) and NfL (β = 0.09, p = 0.0023) compared to those with normal PEF (Table 2B). Beta coefficients represent the standardized difference (in SD units) in biomarker levels between ILF and normal PEF groups. Additional adjustment for systolic blood pressure and blood glucose measured in 2016 did not alter the observed associations.

### Decline in lung function and AD biomarkers

A greater decline in % predicted PEF from 2012/2014 to 2016/2018 was associated with higher levels of neurodegeneration markers in 2016 (Table 2C). Participants in the quartile with the greatest decline in PEF had significantly higher concentrations of p-Tau181 (β = 0.11, p = 0.009), NfL (β = 0.18, p < 0.001), and GFAP (β = 0.09, p = 0.008) compared with those in the quartile with the smallest decline. When modeled continuously, each 1-SD greater decline in PEF was associated with higher levels of p-Tau181 (β = 0.05, p = 0.002), NfL (β = 0.07, p < 0.001), and GFAP (β = 0.05, p = 0.0002). No significant associations were observed for the Aβ42/40 ratio.

### Impaired lung function, AD biomarkers and risk of dementia:

Individuals with impaired lung function (ILF: % predicted PEF < 80) had higher prevalence of dementia (OR=1.63, 95%CI = [1.04, 2.55]; p=0.0300) and CIND (OR=1.82, 95%CI = [1.47, 2.25]; p <.0001) in 2014 compared to those with normal PEF (% predicted PEF ≥ 80) after adjustment for all covariates. Among those participants without dementia in 2014 (n=3969, combined Normal and CIND), 6.9% (n=272) developed dementia over 6 years of follow-up. Individuals with ILF in 2014 had higher risk of developing dementia (HR=1.74, 95%CI = [1.34, 2.25]; p<.0001) compared to those with normal PEF after adjusting for all covariates. Including the AD biomarkers in the model as covariates modestly attenuated the strength of association between ILF and incident dementia (HR=1.67, 95%CI = [1.29, 2.17]; p=0.0001).

### AD biomarkers mediated the association between ILF and incident dementia over 6 years of follow up:

**Figure 2:**
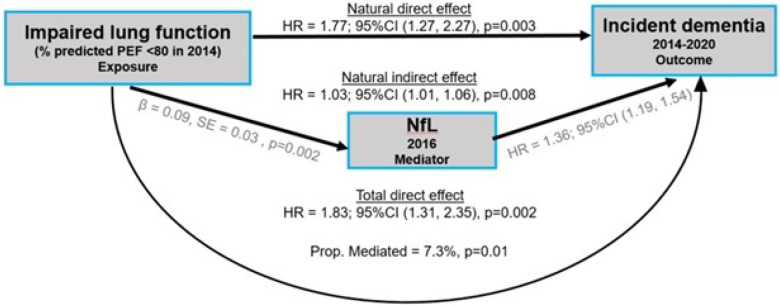
Causal mediation analysis of the association between decline in % predicted PEF and incident dementia mediated by plasma NfL. The path diagram of the causal mediation model with a three-variable system. In Path A, Impaired lung function ILF (exposure) has a significantly positive relationship with NfL (mediator) in a multivariable linear regression model. In Path B, ILF (exposure) was shown as an independent predictor for incident dementia (outcome) in the multivariable Cox hazards regression model without NfL. In Path C, both ILF (exposure) and NfL (mediator) remained significant to predict incident dementia using the multivariable causal mediation model with NfL partially mediate the association between ILF and incident dementia. HR: hazard ratio; CI: confidence interval, β: regression coefficient.

Causal mediation analysis was conducted to evaluate the role of AD biomarkers in the association between baseline impaired lung function (ILF) and incident dementia over a 6-year follow-up period among 3969 individuals without dementia in 2014 survey. Among the AD biomarkers associated with baseline ILF, plasma NfL and serum p-Tau 181 demonstrated partial mediation of this relationship in independent causal mediation models. Plasma NfL accounted for 7.3% of the total association between ILF and dementia (p = 0.01; Figure 2), while serum p-Tau181 mediated 4.9% of the association (p = 0.05; figure not shown) after accounting for baseline age, sex, race, BMI, smoking status, education, comorbidity index and *APOE e4*.

In a sensitivity analysis using dementia follow-up from 2016, we evaluated the mediation effect of NfL. The estimated proportion of the effect mediated increased slightly from 7.3% to 8.1%, although its statistical significance was attenuated (p-value increased from 0.01 to 0.07). Despite this, both the natural direct effect (OR = 1.55, p = 0.049) and the natural indirect effect (OR = 1.03, p = 0.044) remained statistically significant.

## DISCUSSION

In this study of older adults from the Health and Retirement Study, impaired lung function was associated with elevated levels of key AD blood biomarkers including NfL and p-Tau 181 and increased risk of developing dementia over a 6-year follow-up period. To our knowledge, this is the first study to establish an association between impaired lung function and circulating AD protein biomarkers. Notably, we found that plasma NfL and serum p-Tau 181 partially mediated the association between baseline impaired lung function and future risk of dementia, suggesting a potential neurodegenerative pathway linking respiratory dysfunction to cognitive decline.

Blood biomarkers of AD/ADRD have gained significant attention in recent years due to their potential clinical utility in early identification and risk classification for neurodegeneration, dementia, and ultimately, Alzheimer’s disease^13,19^. Previous studies of AD biomarkers demonstrated that the cardiovascular and metabolic risk factors including BMI, renal function and vascular risk factors such as hypertension and diabetes affect the distribution of levels of AD biomarkers in blood^34,20,35^. Our study marks the first attempt to investigate the effect of lung function on blood biomarkers of AD. We demonstrated that lower baseline PEF is associated with higher levels of NfL and p-Tau 181 after two-four years of follow-up. Additionally, a greater decline in PEF over 2 years is associated with elevated levels of NfL, p-Tau 181 and GFAP, indicating that respiratory health may contribute to neurodegenerative pathology. Prior cohort studies have established a link between impaired lung function and neuropathological changes, such as reduced brain volume and increased white matter lesions, suggesting potential mechanisms through which respiratory health may influence future cognitive decline^36–38^. Our findings extend this evidence by demonstrating a link between impaired lung function and elevated levels of blood biomarkers of neuropathology, suggesting that neurodegenerative pathways linking impaired respiratory health and greater risk of dementia. Consistent with our results, a meta-analysis reported that lower FEV_1_ and FVC were significantly associated with reduced neuroimaging markers of brain integrity, including total brain, gray matter, and hippocampal volumes, as well as greater white matter hyperintensity burden^11^. A large longitudinal study in the UK Biobank also established association of restrictive and obstructive impairment in lung function with all-cause dementia and brain MRI structural features of dementia^5^.

We found that baseline impaired lung function is associated with higher odds of having CIND and dementia. In our study, individuals with impaired lung function (PEF < 80%) had a 74% higher risk of developing dementia over a six-year follow-up period. Our findings are consistent with several reports of an association between better lung function and reduced dementia rate in other cohort studies^1,6,10^. Investigations in a younger cohort in the ARIC study with a longer follow-up also showed that individuals with impaired baseline lung function and restrictive/obstructive lung diseases have higher odds of cognitive impairment and dementia in later life^8,9^. Lutsey et al.^9^ reported that restrictive lung diseases, including idiopathic pulmonary fibrosis, were associated with a 58% increased risk of dementia or mild cognitive impairment (MCI), while obstructive lung diseases, such as COPD, were linked to a 33% higher risk. Another recent study in the ARIC cohort by Shrestha et al. with extended follow-up data reported that better lung function–measured by FEV_1_, FVC, and FEV_1_/FVC ratio–was associated with a slower cognitive decline across multiple domains and reduced dementia rate^7^. Additionally, prospective analyses from the CARDIA study, which followed participants from young adulthood to midlife, demonstrated that lower cumulative pulmonary function (FEV_1_ and FVC measured repeatedly over 20 years) was associated with higher midlife cognitive performance. Specifically, cumulative FEV_1_ and FVC were linked to better executive function (Stroop test) and psychomotor speed/attention (Digit Symbol Substitution Test (DSST)), even after adjusting for age, sex, race, smoking, and comorbidities. Notably, lower cumulative FEV_1_ also showed a marginal association with higher verbal memory (RAVLT), suggesting lung health may differentially impact cognitive domains^4^. The Rotterdam Study showed that the FVC but not FEV_1_ or ratio (PRISm (FEV_1_/FVC≥70% and FEV_1_ < 80% predicted)) to be associated with dementia, independent of COPD^1^. They found that participants with FVC % predicted values in the lowest quartile compared to those in the highest quartile were at increased risk of all cause dementia (adjusted HR = 2.28; 95% CI = 1.31–3.98) and AD (HR = 2.13; 95% CI= 1.13–4.02), but no significant association was observed between FEV_1_ and FEV_1_/FVC ratio with incident all cause dementia or AD^1^. These findings highlight that early-life impaired lung health, particularly restrictive lung function, increases susceptibility to cognitive impairment and dementia.

We demonstrated, for the first time, that plasma neurofilament light (NfL) and serum phosphorylated tau 181 (p-Tau 181) were identified as partial mediators, accounting for 7.3% and 5% of the association between impaired lung function and dementia risk, respectively, suggesting a potential biological pathway linking ILF to neurodegeneration. Though AD biomarkers measured concurrently when follow-up of dementia started. We performed sensitivity analysis following up participants after AD biomarker measures and observed that impaired lung function associated with incident dementia and NfL moderately mediated the association. These findings add to the growing body of evidence linking respiratory health to cognitive decline. A study examining the correlation between physical activity, serum NfL concentration, and cognitive decline found that participants with high levels of serum NfL who engaged in medium and high physical activity had a slower rate of cognitive decline compared to those with low physical activity^39^. This might suggest the potential influence of physical activity on improved lung function in mitigating the impact of Alzheimer’s disease pathology on cognitive function. Also, previous studies indicate that chronic hypoxia from respiratory illnesses such as COPD and sleep apnea can cause cognitive deficits, affecting attention, memory, and executive function^40^. This evidence highlights the need for clinical assessment of patients with lung function decline or COPD who have symptoms of neurodegeneration^41^. Evidence from a recent study on COVID-19 patients showed that higher GFAP levels at follow-up were associated with mild cognitive dysfunction. Since COVID-19 primarily affects respiratory function, its long-term impact on neuroinflammation and neurodegeneration has raised concerns about its potential role in Alzheimer’s disease (AD) development^42,43^. In our study, we observed that AD-related biomarkers–particularly those reflecting general neurodegeneration–partially mediated the relationship between impaired lung function and increased dementia risk in older adults. These findings are consistent with a hypothesized pathway in which lung impairment contributes to neurodegenerative processes, possibly through mechanisms involving hypoxia and systemic inflammation^20,44^. However, the complexity of these interactions suggests that additional factors may be involved, warranting further investigation to fully understand the underlying mechanisms. These findings highlight the importance of early detection of cognitive impairment through blood-based biomarkers in individuals with impaired lung function.

### Strengths and limitations:

A major strength of this study is the availability of repeated measures of lung function, and cognitive function in a nationally representative sample of older adults, along with interim AD biomarker assessments. Additionally, the study enhances the robustness of the findings by effectively controlling for multiple confounding variables associated with both AD protein biomarkers and lung function. However, there are several limitations. First, only PEF was available as a measure of lung function, which may not fully capture respiratory impairment. Future studies incorporating more sensitive measures, such as FEV_1_ and FVC, are warranted. Second, AD biomarkers were measured at a single time point, limiting the ability to assess the longitudinal relationship between lung function decline and changes in AD biomarker levels. Third, in this study we investigated only four key AD-related biomarkers (Aβ42/40, p-Tau 181, GFAP, and NfL), which, while informative, do not capture the full spectrum of vascular dysfunction, neuroinflammation, or other potential pathways linking respiratory health to dementia. Future research should incorporate a broader panel of biomarkers, including markers of endothelial dysfunction, systemic inflammation, and cerebrovascular health, to better characterize the biological mechanisms underlying this association.

### Conclusion

In the Health and Retirement Study, impaired lung function was associated with elevated levels of key neuropathology biomarkers in blood, with NfL and p-Tau 181 partially mediating its association with risk of dementia. Our study findings highlight the importance of monitoring AD protein biomarkers in individuals with impaired respiratory health, which may help identify those at higher risk for cognitive decline and support timely interventions to mitigate neurodegenerative processes. These results warrant the need for further research to explore additional molecular biomarkers that mediate the association between impaired respiratory health and future risk of cognitive impairment and dementia.

## Figures and Tables

**Table 1:** Descriptive statistics of participant characteristics in the HRS 2012/2014 survey across lung function groups

Estimates Mean ± SD / Frequency (%)	Overall n = 4072	Normal (Predicted PEF ≥ 80%) n = 3191 (78.4%)	Impaired lung function (Predicted PEF < 80%) n = 881 (21.6%)	p value
% predicted PEF 2012/2014	98.96 ± 25.74	108.65 ± 19.08	63.85 ± 13.03	<.0001
Age 2014 (years)	66.19 ± 10.29	66.32 ± 10.26	65.70 ± 10.38	0.11
Sex (% females)	2397 (58.9%)	1891 (59.3%)	506 (57.4%)	0.3300
Race/ethnicity	667 (16.4%)	499 (15.6%)	168 (19.07%)	0.0003
Blacks	601 (14.8%)	453 (14.2%)	148 (16.8%)	
Hispanics	129 (3.2%)	91 (2.9%)	38 (4.3%)	
Other	2675 (65.7%)	2148 (67.3%)	527 (59.8%)	
Whites				
Smoking status 2014	501 (12.3%)	284 (8.9%)	217 (24.6%)	< .0001
Current	991 (24.3%)	790 (24.8%)	201 (22.8%)	
Former	2580 (63.4%)	2117 (66.3%)	463 (52.6%)	
Never				
Education	747 (18.3%)	503 (15.8%)	244 (27.7%)	< .0001
0–11 y	1252 (30.8%)	944 (29.6%)	308 (35.0%)	
12 y	1023 (25.1%)	843 (26.4%)	180 (20.4%)	
13–15y	1050 (25.8%)	901 (28.2%)	149 (16.9%)	
16 + y				
Body Mass Index (kg/m^2^) 2012/2014	30.56 ± 6.91	30.65 ± 6.74	30.27 ± 7.50	0.1400
Comorbidity Index 2014	1.88 ±1.29	1.83 ± 1.26	2.06 ± 1.36	< .0001
eGFR (Cys and CR) 2016	68.79 ±21.08	68.61 ± 21.17	69.44 ± 20.76	0.3100
Inflammatory latent variable 2016	0.08 ± 0.51	0.09 ± 0.51	0.04 ± 0.49	0.0060
APOE e4 allele, %Yes	1122 (27.6%)	898 (28.1%)	224 (25.4%)	0.1100
Cognitive function 2014 (0–27)	15.55 ± 4.22	15.89 ± 4.14	14.32 ± 4.28	< .0001
Cognition category in 2014	3388 (83.2%)	2742 (85.9%)	646 (73.3%)	< .0001
Normal	581 (14.3%)	380 (11.9%)	201 (22.8%)	
CIND	103 (2.5%)	69 (2.2%)	34 (3.9%)	
Dementia				
Incident dementia* n = 3969	272 (6.9%)	172 (5.5%)	99 (11.6%)	< .0001

Note:

* Dementia was based on a cognitive score of 1–6 in 2016, 2018 or 2020 among participants who had normal cognition or CIND in 2014.

**Table 2 A:** Association between percent predicted PEF in 2012/2014 (baseline) and blood biomarkers of AD in 2016

AD biomarkers	Q1 β (SE), p value	Q2 β (SE), p value	Q3 β (SE), p value	Q4	1 SD unit β (SE), p value
Aβ 42/40 ratio ~ % pred PEF	0.04 (0.05), 0.42	0.03 (0.05), 0.48	0.008 (0.04), 0.86	Reference	−0.01 (0.02), 0.4600
p-Tau 181 ~ % pred PEF	0.15 (0.04), 0.0002	0.09 (0.04), 0.03	0.11 (0.04), 0.007	Reference	−0.04 (0.01), 0.0040
NfL ~ % pred PEF	0.11 (0.03), 0.0006	0.09 (0.03), 0.005	0.02 (0.03), 0.63	Reference	−0.05 (0.01), < .0001
GFAP ~ % pred PEF	0.10 (0.03), 0.004	0.09 (0.03), 0.008	0.07 (0.03), 0.03	Reference	−0.04 (0.01), 0.001

Note: AD biomarkers were log transformed and standardized to approximate the normal distribution.

Models were adjusted for age, sex, race, education, BMI, smoking status, comorbidity index 2014, inflammatory latent variable 2016, eGFR 2016, and APOE e4 allele.

**Table 2 B:** Association between impaired lung function in 2012/2014 and blood biomarkers of AD in 2016

	β (SE), p value
Aβ 42/40 ratio	0.03 (0.04); 0.52
p-Tau 181	0.10 (0.03); 0.004
NfL	0.09 (0.03); 0.002
GFAP	0.04 (0.03); 0.22

Note: AD biomarkers were log transformed and standardized to approximate the normal distribution.

Models were adjusted for age, sex, race, education, BMI, smoking status, comorbidity index, inflammatory latent variable, eGFR, and APOE e4 allele.

**Table 2 C:** Association of decline in % predicted PEF from 2014 to 2016 as quartile and continuous variable with blood biomarkers of AD in 2016

AD biomarkers	Q1	Q2 β (SE), p value	Q3 β (SE), p value	Q4 β (SE), p value	1 SD unit β (SE), p value
Aβ 42/40 ratio	Reference	−0.02 (0.05), 0.69	−0.04 (0.05), 0.42	−0.03 (0.05), 0.51	−0.003 (0.02), 0.8500
p-Tau 181	Reference	−0.02 (0.04), 0.59	0.08 (0.04), 0.07	0.11 (0.04), 0.009	0.05 (0.02), 0.0020
NfL	Reference	0.05 (0.03), 0.12	0.08 (0.03), 0.02	0.18 (0.03), < .0001	0.07 (0.01), < .0001
GFAP	Reference	−0.06 (0.03), 0.08	0.01 (0.03), 0.86	0.09 (0.03), 0.008	0.05 (0.01), 0.0002

Note: AD biomarkers were log transformed and standardized to approximate the normal distribution.

Models were adjusted for % predicted PEF in 2014 (baseline), age, sex, race, education, BMI, smoking status, comorbidity index 2014, inflammatory latent variable 2016, eGFR 2016, and APOE e4 allele.

## Data Availability

We used the HRS publicly available datasets and sensitive biomarker data for this study analysis. This data can be found here: https://hrsdata.isr.umich.edu/data-products/public-survey-data and https://hrsdata.isr.umich.edu/dataproducts/sensitive-health and can be accessed by completing required data use agreement.
